# Current News and Opinion

**Published:** 1885-04

**Authors:** 


### HYDROCHLORATE OF COCAINE.

For the last few months every medical and dental journal in the land, and nearly every newspaper published, has expatiated to a greater or less extent on the merits of this newly discovered pain-obtunding agent, and wonderful effects have been reported in a great variety of cases where it has been employed. In our dental society gatherings it has proved a theme for much speech-making, and if all that has been stated concerning its practical value in the many cases cited is absolutely "unvarnished truth," the joyous shout of "Eureka" might well resound from every human throat.

But are we not sometimes a little hasty in jumping at conclusions, and do we not allow ourselves to be so overcome with enthusiasm over newly-discovered remedies and appliances that we pass judgment on half trials, and suffer our imagination to fill out the blank?

There is not a doubt that cocaine is an exceedingly valuable agent as a pain obtunder, and is destined to act an important part in the field of surgery, but in our specialty its use must be somewhat limited. To be sure, reports have been heralded from every quarter concerning its marvelous action in all sorts of cases where it has been applied, but we fear that some of the reports are somewhat tinged with "fancy" coloring.

We notice also, that in the hands of different operators like results are not always secured. Some have applied it to sensitive dentine, and report that in every case it has rendered the operation of preparing cavities painless. Others have experimented with great care in the same direction, but with no success. According to reports, the same diverse results have followed where this agent has been applied to exposed and aching pulps, as well as in cases of peridontitis and facial neuralgia. And where a four per cent. solution has proved valueless in some of these cases, other remedies, long in use, have worked nicely.

Several of our enthusiastic friends, failing to secure the desired result in applying cocaine to sensitive dentine, have tried hypodermic injections within the oral cavity, with a view of causing temporary paralysis of the maxillary nerves; but could this be accomplished, such treatment would be far from practicable. Few would submit to it, and few cases would warrant such means.

C. E. F.

### PRESERVING TOOTH PULPS.

Apropos of the recent articles on "Capping Exposed Pulps," in the INDEPENDENT PRACTITIONER, I will write you of a method, which, so far as I know, has never been published or practiced except by myself. We are often called upon to fill large approximal cavities in bicuspids and the crowns of molars. The method which I use in such cases, though not strictly "capping," still is sufficiently so to come under that heading. I first cover that portion of the cavity next the pulp with a layer of oxy-phosphate, with which a very small quantity of iodoform has been mixed. This medicated layer of oxy-phosphate has a direct prophylactic influence, not only on the layer of semi-decayed dentine under it, but also on the pulp itself, and seems to stimulate and assist it to recuperative action as well as to prevent any putrescent tendency, and this feature constitutes its peculiar characteristic. In order that no undue pressure may be made on the pulp, I mix the oxy-phosphate quite thin, and carry it to place with an instrument on which a small quantity of cotton is wound loosely. After this layer has set, I fill the rest of the cavity with a temporary filling of oxy-phosphate, in the usual way.

I am aware that carbolic acid and creosote have been used in this manner, and for this purpose; in fact, I have tried nearly everything that has been recommended, but I have never heard that iodoform has been so employed. I have been practicing this method for some time, and on all sorts of cases, in some of which the pulp was actually bare, and so far as I know, I have not had a failure.

For the sake of testing this idea (for I think it is worth testing), I wish that others would experiment with it, and let us know the results.

D. W. B

---

### ILLINOIS STATE DENTAL SOCIETY.

The twenty-first annual meeting of the Illinois State Dental Society will be held at Peoria, Illinois, commencing Tuesday, May 12, 1885, and continuing four days.

The State Board of Dental Examiners will meet at the National Hotel at 10 A. M., Monday, May 11th, at which time candidates for examination must present themselves punctually. The examinations will occupy until Thursday, May 14th.

J. W. WASSALL, Secretary,
103 State St., Chicago.

### NEW YORK COLLEGE OF DENTISTRY.

The nineteenth annual commencement of the New York College of Dentistry was held at Chickering Hall, Fifth Avenue and Eighteenth Street, on Monday evening, March 9, 1885.

The degree of Doctor of Dental Surgery was conferred upon forty-six candidates by M. McN. Walsh, Esq., President of the Board of Trustees. The valedictory was delivered by Louis A. Queen, D. D. S., and the address to the graduates by J. Smith Dodge, M. D., D. D. S. The number of matriculates is one hundred and sixty-five.

The graduates are as follows:

| | | | |
|---|---|---|---|
| Allen, Fremont | N. Y. | Hawes, Arthur F | N. J. |
| Allen, Lyndon C | N. Y. | Kleinhans, Daniel W | N. J. |
| Arndt, Louis | N. J. | Knox, Leonard K | N. Y. |
| Atkinson, Wm. F | N. Y. City. | King, Lyman S | N. Y. City. |
| Beldad, Enrique G. de la | Spain. | McDougal, Allan S | N. Y. |
| Beldad, Ramon G. de la | Spain. | Mooney, Chas. J | N. Y. |
| Blair, Lemuel P | Maine. | Moore, Joseph W | N. Y. City. |
| Brinkman, Max R | Mass. | Morse, Wellslake D | N. Y. City. |
| Cady, Herbert W. F | N. Y. City. | O'Brien, Henry L | N. Y. |
| Cochran, Robert H | Mass. | Pape, Frederick W | Germany. |
| Crane, Frank S | N. J. | Parker, Virgil F | N. Y. |
| Degenhardt, Martin | N. Y. City. | Queen, Louis A | N. J. |
| Dodge, Arthur | Conn. | Rivinius, Julius W | N. Y. City. |
| Dunges, John | Germany. | Roberts, Livingston J | N. Y. City. |
| Eagleton, Charles H | N. Y. | Roberts, John | England. |
| Fitzpatrick, Thos. A | N. Y. City. | Shenstone, Joseph N | N. Y. |
| Fonda, Edw. S., M. D | N. Y. City. | Simon, Samuel | Mass. |
| Gibbs, Frederick W | Conn. | Smith, Karl C | N. Y. |
| Henriquez, Joseph M | W. I. | Smith, Charles R | N. J. |
| Hull, Henry J | N. Y. City. | Swift, Arthur L | N. J. |
| Herrera, Ignacia V | Cuba. | Weber, Johan A. Theo | Finland. |
| Harnung, George J | N. J. | Wyant, Wm. M | N. Y. |
| Howard, Chas. W | N. Y. | Villalon, Louis M | Porto Rico. |

---

### THE ODONTOLOGICAL DINNER.

A full stenographic report of everything said at the annual dinner of the New York Odontological Society was made at the time, and it will be published in the *Odontographic Journal* for April. The proceedings were very interesting, and some of the speeches were capital.

---

### UNIVERSITY OF MARYLAND, DENTAL DEPARTMENT.

The annual commencement of 1885, in connection with the seventy-eighth annual session, was held at the Academy of Music, Baltimore,

Tuesday, March 17th. Prayer by Rev. Culbreith B. Perry. Reading of mandamus and announcement of graduates by the dean, Professor F. J Gorgas. Conferring of degrees and award of prizes by Hon. S. Teackle Wallis, LL. D., Provost of the University. Address by Prof. R. Dorsey Coale, Ph. D.

Graduates in dental surgery, thirty-six in number:

| | |
|---|---|
| Bailey, Madison A. . . . . . . . . . . . . S. C. | Josselyn, Eli E., M. D . . . . . . . . N. B. |
| Beadles, E. Payson . . . . . . . . . . . . . . Va. | Kloeber, John S. . . . . . . . . . . . . . . . Va. |
| Bradford, Henry Clinton. . . . . . . . . Va. | Matthews, Augustus. . . . . . . . . . . N. C. |
| Brown, Claude D. . . . . . . . . . . . . . . Va. | McQuown, Robert T. . . . . . . . . . . . . Va. |
| Carlisle, John P. . . . . . . . . . . . . . . . S. C. | McQuown, William P. . . . . . . . . . . Va. |
| Carter, Joseph W. . . . . . . . . . . . . . . Mo. | Norwood, Wm. McIntosh. . . . . . S. C. |
| Comegys, Thomas M. . . . . . . . . . Tenn. | Parker, Will W. . . . . . . . . . . . . . . Minn. |
| Cooke, Frank J. . . . . . . . . . . . . . . . . Tex. | Pitts, Henry Clay . . . . . . . . . . . . . N. C. |
| Dorset, Willie Edward. . . . . . . . . . . Va. | Perkins, Capers D. . . . . . . . . . . . . . . Ga. |
| Fournier, Joseph, Jr. . . . . . . . . . . N. Y. | Ranson, James M., Jr. . . . . . . . W. Va. |
| Groshans, Ferdinand . . . . . . . . . . . . Md. | Rutledge, Brooks. . . . . . . . . . . . . . S. C. |
| Hebbel, Charles W. . . . . . . . . . . . . . Md. | Schaer, Charles T. . . . . . . . . . . . . . . Md. |
| Helm, John W. . . . . . . . . . . . . . . . . . Md. | Trapp, Wm. Sherman. . . . . . . . . . . . Pa. |
| Hill, Charles E. . . . . . . . . . . . Australia. | Twitchell, Fred. A. . . . . . . . . . . . . Minn. |
| Houghland, Ulysses Sylvester. . . . Ind. | Wangemann, Albert. . . . . . . Germany. |
| Howland, Clarence Henry. . . . . . D. C. | Welch, Floyd J. . . . . . . . . . . . . . . . . Va. |
| Howlett, A. Hersey. . . . . . . . . . . . . . Pa. | Wegge, William F. . . . . . . . . . . . . . Wis. |
| Hundley, Peyton. . . . . . . . . . . . . . . . Va. | Wood, Frank Le Roy. . . . . . . . . Maine. |

Number of matriculates for session of 1884–85, seventy-four.

---

### KANSAS STATE DENTAL LAW.

Spread the news throughout the land that Kansas keeps step with the march of progress. On February 25th its Legislature enacted a law regulating the practice of dentistry within its borders. In its action it is identical with the law now in force in Missouri, allowing none but graduates of reputable dental schools to engage in the practice of dentistry in the State in the future, and a knowledge of the men most active in its enactment warrants us in saying that it will be rigidly enforced. Dr. L. C. Wasson, of Ottawa, Kansas, who is a member of the State Senate, introduced the bill, and to his ability and influence is largely due its successful passage, and it will always remain as a fitting monument to his love and regard for the dignity and character of his chosen calling. May he live long to enjoy its fruits.

The next annual meeting of the Kansas State Dental Society will be held at Topeka, Kansas, commencing Tuesday, May 5, 1885, and the indications now point to the largest gathering in the history of the society. DENTOS.

### THE MEDICAL CONGRESS OF 1887.

The organization of the section of Dental and Oral Surgery in the International Medical Congress, that will meet in Washington in 1887, has been completed by the appointment of the following gentlemen as officers:

*President*—J. Taft, M. D., D. D. S., Cincinnati, Ohio.

*Vice-Presidents*—W. W. Allport, M. D., D. D. S., Chicago, Ills.; W. H. Dwinelle, M. D., D. D. S., New York City; J. L. Williams, M. D., D. D. S, Boston, Mass.

*Secretaries*—E. A. Bogue, M. D., D. D. S., New York City; G. H. Cushing, D. D. S., Chicago, Ills.

*Members of Council*—W. C. Barrett, M. D., D. D. S., Buffalo, N. Y.; Thos. Fillebrown, M. D., D. M. D., Portland, Me.; F. J. S. Gorgas, M. D., D. D. S., Baltimore, Md.; Ed. Maynard, M. D., Washington, D. C.; J. H. McKellops, D. D. S., St. Louis, Mo.; W. H. Morgan, M. D., D. D. S., Nashville, Tenn.; C. N. Pierce, D. D. S., Philadelphia, Pa.; L. D. Shepard, D. M. D., Boston, Mass.; James Truman, D. D. S., Philadelphia, Pa.; J. W. White, M. D., D. D. S., Philadelphia, Pa.

---

### SECOND DISTRICT DENTAL SOCIETY.

At the annual meeting of the Second District Dental Society, held on Monday, March 9th, at the office of Dr. L. G. Wilder, 54 Fort Greene Place, Brooklyn, the following officers were elected:

*President*—E. Parmly Brown, Flushing, L. I.

*Vice-President*—C. W. Harreys, Brooklyn, N. Y.

*Recording Secretary*—A. N. Roussel, 879 Gates Ave., Brooklyn.

*Corresponding Secretary*—F. T. Van Woerl, Brooklyn, N. Y.

*Treasurer*—L. G. Wilder, Brooklyn, N. Y.

*Librarian*—D. S. Skinner, Brooklyn, N. Y.

A. N. ROUSSEL, Rec. Sec.

---

### FIFTH DISTRICT DENTAL SOCIETY.

The Fifth District Dental Society of the State of New York will hold its seventeenth annual meeting at Utica, Tuesday and Wednesday, April 7 and 8, 1885. The session will be called to order at two P. M., at the Butterfield House.

### DENTAL LEGISLATION IN DAKOTA.

The legislative assembly of Dakota has passed an admirable dental law. Profiting by the mistakes of some of the older States the dentists of that Territory drafted a law that seems as near perfect as they could hope for. One excellent provision is that all registrations shall be made under oath. The diplomas of colleges, also, require the indorsement of the examining board, thus preventing practice under a diploma illegally obtained, and allowing the board to throw out those of any fraudulent, or disreputable, or incompetent college.

---

### SEVENTH DISTRICT DENTAL SOCIETY.

The seventeenth annual convention of the Seventh District Dental Society of the State of New York will be held in Rochester, on Tuesday and Wednesday, April 28 and 29, 1885. Members of the profession are invited to be present.

CHAS. T. HOWARD, Recording Secretary,
224 E. Main St., Rochester, N. Y.

---

### NEBRASKA STATE DENTAL SOCIETY.

The ninth annual meeting of the Nebraska State Dental Society will meet at Lincoln, Tuesday, May 12, 1885, and continue in session three days.

W. F. ROSEMAN, Secretary,
Fremont, Nebraska.

---

### EIGHTH DISTRICT DENTAL SOCIETY.

The seventeenth annual meeting of the Eighth District Dental Society of the State of New York will be held in the City of Buffalo, commencing on Tuesday, April 21st, 1885, and will continue two days.

---

### THE INTERNATIONAL TOOTH CROWN COMPANY.

Too late for insertion in this number, we have received a communication in answer to the article of Dr. Dwinelle, in the number for October, 1884. It shall have attention next month.

(51.) Will tin or amalgam placed at the cervical wall of proximal cavities of bicuspids and molars, preserve those margins better than gold, provided equal care and skill is used in each case? And if so, why? A. B. M.

(52.) Will some one kindly give the formula for making "Acid Sulph. Arom." in the next I. P., and greatly oblige, R. C. M.

---

### Answers.

R. C. M. (52.)

| | | |
|---|---|---|
| R. | Acid Sulph. (pure), | oz. vj. |
| | Zinzib. Rad. (Jamaica), | oz. i. |
| | Cinnam. Cort. | oz. iss. |
| | Sp. Vin. Rec., | q. s. |

Add the acid gradually to a pint of alcohol and allow it to cool. Mix together the ginger and cinnamon, reduced to a coarse powder, and place them in a percolator. Pour alcohol gradually upon them until a pint of tincture is obtained, and mix the diluted acid with this tincture. C. E. F.

B. (49.) By the term "knuckling," it is intended to convey the close approximation of adjoining teeth or fillings, at the small point of contact just above the inter-dental portion of the festoon of gum surrounding the necks of the teeth. The simplest way to illustrate this form, being nature's best, is to place the backs of the hands together on a table, with the knuckles on the table about one inch apart, the gum being supposed to occupy the open space from the table to the point of union of the backs of the hands near the wrists. These forms in nature resist decay the best, and keep clean and comfortable, both in nature and art. E. Parmly Brown.

B. (49.) The term "knuckling" signifies the complete and perfect restoration of the approximal side of a tooth, with any filling material, to its original type. When this is done, there is a point of contact (the knuckle) between the teeth, which prevents the crowding of food down on the gum, and also helps to strengthen the dental arch as each tooth is leaning against its neighbor.
A. N. Roussel.

Student. (41.) Take waste amalgam, old fillings, etc., put them into a crucible without flux; increase the temperature to a *red* heat, and continue the same about ten minutes; pour the alloy upon a flat surface. This process reduces the weight a little more than one-half, from loss of mercury and other volatile metals. Weigh the ingot thus obtained, and to eighty parts of the alloy add seventeen parts fine silver and three parts fine gold. The silver may be readily obtained of a silver-plater. For convenience, both silver and gold should be rolled into thin plate, and be cut into strips. Select a crucible of liberal size for the quantity to be melted, in which fuse borax so that the molten salt will fill the crucible at least one-third full. Add piece by piece of the broken ingot till all is fused, then add the silver strips, which will immediately disappear, and at last the gold; this too will as quickly be melted, no greater heat being required than is necessary to render the flux fluid. Stir the alloy well with a pipe stem, or a clean iron wire, and pour into any shallow mould which will give form to the alloy most convenient for cutting. Although this alloy must be of unknown quantities, practical experiments prove it to be of a superior quality, of good color, and quick setting. Full instructions for making various grades of amalgam, its adaptation to conditions, etc., may be found in a work on "Plastics and Plastic Fillings," by J. Foster Flagg, sold by the S. S. White Dental Manufacturing Co. S. B. P.

